# Antibacterial Activity and Components of the Methanol-Phase Extract from Rhizomes of Pharmacophagous Plant *Alpinia officinarum* Hance

**DOI:** 10.3390/molecules27134308

**Published:** 2022-07-05

**Authors:** Junfeng Fu, Yaping Wang, Meng Sun, Yingwei Xu, Lanming Chen

**Affiliations:** 1 Key Laboratory of Quality and Safety Risk Assessment for Aquatic Products on Storage and Preservation (Shanghai), Ministry of Agriculture and Rural Affairs of the People’s Republic of China, College of Food Science and Technology, Shanghai Ocean University, Shanghai 201306, China; m190300718@st.shou.edu.cn (J.F.); m210311022@st.shou.edu.cn (M.S.); m190300760@st.shou.edu.cn (Y.X.); 2 Department of Internal Medicine, Virginia Commonwealth University/McGuire VA Medical Centre, Richmond, VA 23298, USA; yapingwang2016@gmail.com

**Keywords:** *Alpinia officinarum* Hance, rhizome, antibacterial activity, antibacterial compound, transcriptome

## Abstract

The rhizomes of *Alpinia officinarum* Hance (known as the smaller galangal) have been used as a traditional medicine for over 1000 years. Nevertheless, little research is available on the bacteriostatic activity of the herb rhizomes. In this study, we employed, for the first time, a chloroform and methanol extraction method to investigate the antibacterial activity and components of the rhizomes of *A. officinarum* Hance. The results showed that the growth of five species of pathogenic bacteria was significantly inhibited by the galangal methanol-phase extract (GMPE) (*p* < 0.05). The GMPE treatment changed the bacterial cell surface hydrophobicity, membrane fluidity and/or permeability. Comparative transcriptomic analyses revealed approximately eleven and ten significantly altered metabolic pathways in representative Gram-positive *Staphylococcus aureus* and Gram-negative *Enterobacter sakazakii* pathogens, respectively (*p* < 0.05), demonstrating different antibacterial action modes. The GMPE was separated further using a preparative high-performance liquid chromatography (Prep-HPLC) technique, and approximately 46 and 45 different compounds in two major component fractions (Fractions 1 and 4, respectively) were identified using ultra-HPLC combined with mass spectrometry (UHPLC-MS) techniques. o-Methoxy cinnamaldehyde (40.12%) and p-octopamine (62.64%) were the most abundant compounds in Fractions 1 and 4, respectively. The results of this study provide data for developing natural products from galangal rhizomes against common pathogenic bacteria.

## 1. Introduction

Traditional pharmacophagous plants are a very good source for obtaining a variety of bioactive compounds and drugs [1]. These compounds are characterized by safety and low toxicity, which allows for their clinical application in the prevention and long-term treatment of human diseases [2]. One such herb is *Alpinia officinarum* Hance that is known as the smaller galangal. *A. officinarum* Hance belongs to the Zingiberaceae family and is widely cultivated in southern China and many Asian countries [3]. The rhizomes of *A. officinarum* Hance have been used as a traditional medicine to relieve stomachache, to invigorate circulation, treat colds, and to reduce swelling for over 1000 years. They are also used as a folk medicine to treat catarrh, bronchial ulcers, and throat infection [4]. However, the bioactive compounds in the rhizomes of *A. officinarum* Hance remain largely unexplored.

Previous studies have revealed three major groups of chemical constituents isolated from the rhizomes of the smaller galangal, including flavonoids, glycosides and diarylheptanoids [5]. Recently, pharmacological activities of these phytochemicals have been reported, including anti-inflammatory, antioxidant, and anticancer effects [3,6,7]. Nevertheless, the current literature is limited with respect to the bacteriostatic activity of the herb rhizomes. Eumkeb et al. reported that flavonoids (galangin, kaempferide and kaempferide-3-O-b-d-glucoside) isolated from the smaller galangal have the potential to reverse bacterial resistance to amoxicillin in amoxicillin-resistant *Escherichia coli* [5]. Srividya et al. investigated different extraction methods using 50% ethanol with either hot or cold maceration of the rhizomes of *A. officinarum* and found that the former extract contained more phenol and flavonol and showed better antibacterial activity, compared to the cold maceration extract [8]. Zhang et al. reported three new antibacterial active diarylheptanoids isolated in an ethanol extract from *A. officinarum* Hance rhizomes, including 7-(4″,5″-dihydroxy-3″-methoxyphenyl)-1-phenyl-4-heptene-3-one, 1,7-diphenyl-5-heptene-3-one, and 4-phenethyl-1,7-diphenyl-1-heptene-3,5-dione. These compounds showed strong antibacterial activity against Hp-Sydney strains 1 and Hp-F44 with minimum inhibitory concentration (MIC) values of 9–12 μg/mL, and 25–30 μg/mL, respectively [9]. Recently, Lakshmanan et al. reported that the active compound, 1-(3,5-dihydroxyphenyl)-2-(methylamino)ethan-1-one, obtained from a methanol extract of *A. officinarum* inhibited the swarming motility of *Pseudomonas aeruginosa* at 12.5 µg/mL. This inhibition was independent of rhamnolipid production. Real-time PCR analysis showed significant downregulation of virulence-associated genes, including T3SS exoS, exoT and the flagella master regulator fleQ [10].

To address the lack of information in this research area, we, for the first time, used a chloroform and methanol extraction (CME) method to investigate the antibacterial activity and components of the rhizomes of *A. officinarum* Hance. The major objectives of this study were: (1) to determine the antibacterial effects of the galangal chloroform-phase extract (GCPE) and the methanol-phase extract (GMPE) against 20 common species of pathogenic bacteria; (2) to identify major components of the GMPE using ultra-high performance liquid chromatography combined with mass spectrometry (UHPLC-MS) techniques; (3) to investigate possible antibacterial action modes of the GMPE against representative Gram-positive and Gram-negative pathogens by comparative transcriptomics analysis. The results of this study provide data for developing antibacterial medicine and food biopreservatives from the rhizomes of *A. officinarum* Hance against common pathogenic bacteria.

## 2. Results and Discussion

### 2.1. Antibacterial Activity of Chloroform- and Methanol-Phase Extracts from the Rhizomes of A. officinarum Hance

Bioactive substances in the rhizomes of *A. officinarum* Hance were extracted using the CME method (see Materials and Methods); the observed extraction yields of GMPE and GCPE were 23.8% and 17.5%, respectively.

The antibacterial activity of the GMPE and GCPE was determined against 20 species of pathogens using a disc diffusion method; the results are presented in Table 1. When compared with the control groups, the growth of ten species of Gram-negative, and one species of Gram-positive bacteria, were significantly inhibited by the GMPE and/or the GCPE (500 mg/mL) (*p* < 0.05). A maximum diameter of the inhibition zone (DIZ) was observed against *Aeromonas hydrophila* when treated with the GMPE, followed by *Staphylococcus aureus*, and *Vibrio parahaemolyticus* treated with the GCPE and/or GMPE. Different effects of the two extracts were also observed against different strains. The differences in the drug resistance of various strains, such as *S. aureus*, may explain the absence of activity of the same extract in some instances. Although the DIZ values were relatively lower against *V. metschnikovii* treated with these two extracts, they were significantly higher than for the control groups (*p* < 0.05). Additionally, no significant antibacterial activity was observed against the other nine species of bacteria tested in this study.

Previous studies have also reported antibacterial activity of the rhizomes of *A. officinarum* Hance [5,8,9,10,11]. For example, Lee and Rhee reported that the methanol and ethylacetate combination extracts from fresh rhizomes of *A. officinarum* Hance were especially effective against four vancomycin-resistant *Enterococci* strains: *E. faecalis* (K-10-22), *E. faecaium* (K-11-212), *E. faecalis* (K-10-57) and *E. faecalis* (K-10-361), with MIC values of 12.5, 12.5, 6.25 and 25 µg/mL, respectively. The combination was also effective against yeasts, such as *Candida albicans*, *Candida tropicalis* and *Cryptococcus neoformans* [11].

In this study, as shown in Table 1, antibacterial activities against *E. sakazakii*, and *V. parahaemolyticus* were only observed for treatment with the GMPE. Moreover, overall higher bacteriostatic effects of the GMPE were observed, particularly against the pathogen *Aeromonas hydrophila,* with a 1.33-fold larger DIZ observed than for treatment with the GCPE. Therefore, the antibacterial properties and the mechanisms of action of the GMPE were investigated further.

The MIC values of the GMPE were determined against five highly inhibited species using a broth micro-dilution assay; the results are shown in Table 2. After treatment with GMPE at concentrations ranging from 500 to 0.98 mg/mL, the observed MIC values against *A. hydrophila*, *E. sakazakii* CMCC45401, *S. aureus* ATCC8095, *V. metschnikovii* ATCC700040, and *V. parahaemolyticus* ATCC17802 were 1.95 mg/mL, 3.9 mg/mL, 3.9 mg/mL, 7.81 mg/mL, and 3.90 mg/mL, respectively.

Similarly, the observed minimum bactericidal concentration (MBC) values of the GMPE were 3.90 mg/mL, 7.81 mg/mL, 7.81 mg/mL, 15.62 mg/mL, and 7.81 mg/mL against *A. hydrophila*, *E. sakazakii* CMCC45401, *S. aureus* ATCC8095, *V. metschnikovii* ATCC700040, and *V. parahaemolyticus* ATCC17802, respectively (Table 2).

### 2.2. Bacterial Cell Structure Change Mediated by GMPE Treatment

To determine possible mechanisms underlying the bacteriostatic activity of the GMPE, the bacterial cell surface hydrophobicity, membrane fluidity and permeability of the five highly inhibited strains were analyzed, as these are key parameters of bacterial cell response to adverse environments [12].

**Figure 1:**
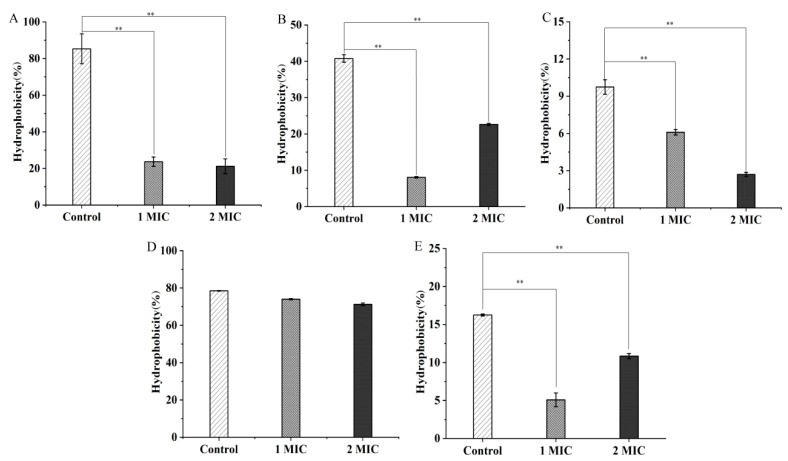
The effects of the GMPE on bacterial cell-surface hydrophobicity. (**A**–**E**) *S. aureus* ATCC8095, *A. hydrophila*, *E. sakazakii* CMCC45401, *V. metschnikovii* ATCC700040, and *V. parahaemolyticus* ATCC17802, respectively. **: *p* < 0.01.

As shown in Figure 1A, when compared with the control groups, the cell-surface hydrophobicity of the Gram-positive bacterium *S. aureus* ATCC8095 was significantly decreased by 3.60- and 4.02-fold after treatment with 1 MIC and 2 MIC of the GMPE at 37 °C for 1 h, respectively (*p* < 0.01). Reduced cell-surface hydrophobicity was also observed in the Gram-negative bacteria *A. hydrophila*, *E. sakazakii* CMCC45401, and *V. parahaemolyticus* ATCC17802. The GMPE treatment possibly affected polar and/or apolar components (such as lipopolysaccharide and proteins) in the outer membrane of the Gram-negative bacteria [13]. For example, after treatment with 1 MIC of the GMPE for 1 h, the cell-surface hydrophobicity of these three strains significantly decreased by 5.05-fold, 1.59-fold, and 3.19-fold, respectively (*p* < 0.01) (Figure 1B,C,E). The higher concentration of the GMPE (2 MIC) enhanced this effect on *E. sakazakii* CMCC45401 (2.26-fold), but opposite profiles were observed for *A. hydrophila* and *V. parahaemolyticus* ATCC17802 (1.80-fold, and 1.50-fold) (*p* < 0.01). Additionally, no significant difference was observed in cell-surface hydrophobicity of *V. metschnikovii* ATCC700040 after the treatments, when compared with the control groups (*p* > 0.05) (Figure 1D).

**Figure 2:**
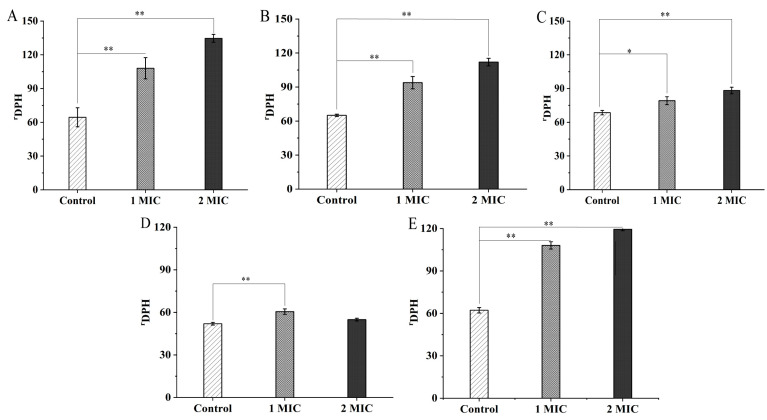
The effects of the GMPE on bacterial cell membrane fluidity. (**A**–**E**) *S. aureus* ATCC8095, *A. hydrophila*, *E. sakazakii* CMCC45401, *V. metschnikovii* ATCC700040, and *V. parahaemolyticus* ATCC17802, respectively. *: *p* < 0.05, and **: *p* < 0.01.

As shown in Figure 2A–E, the GMPE treatments caused a significant increase in the cell membrane fluidity of the five bacterial strains, when compared with the control groups (*p* < 0.05), consistent with their decreased cell-surface hydrophobicity mediated by the GMPE. For example, treatment with 1 MIC for 1 h significantly increased the cell-membrane fluidity of *S. aureus* ATCC8095, *A. hydrophilia,* *E. sakazakii* CMCC45401, *V. metschnikovii* ATCC700040, and *V. parahaemolyticus* ATCC17802 by 1.67-fold, 1.45-fold, 1.15-fold, 1.16-fold, and 1.74-fold, respectively (*p* < 0.05). Moreover, the increased trend in bacterial cell-membrane fluidity was GMPE concentration-dependent (except for *V. metschnikovii* ATCC700040). The changed cell-membrane fluidity possibly resulted in cytoplasmic membrane damage and subsequent cellular content leakage, and even cell death.

**Figure 3:**
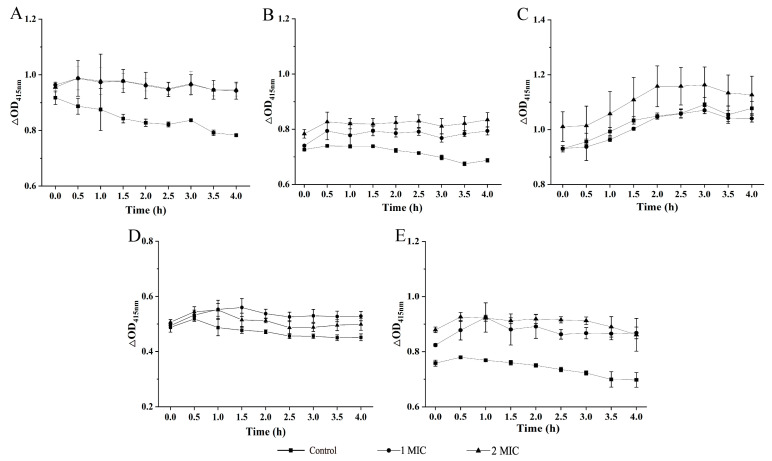
The effects of the GMPE on bacterial CIM permeability. (**A**–**E**) *S. aureus* ATCC8095, *A. hydrophila*, *E. sakazakii* CMCC45401, *V. metschnikovii* ATCC700040, and *V. parahaemolyticus* ATCC17802, respectively.

The bacterial cell membrane is an efficient permeable barrier which can exclude macromolecules and hydrophobic substances [12]. The influence of the GMPE on bacterial cell inner membrane (CIM) permeability was examined using the probe O-nitrophenyl-β-D-galactopyranoside (ONPG); the results are shown in Figure 3A–E. When compared with the control groups, the treatments with 1 MIC or 2 MIC of the GMPE increased CIM permeability of the five bacterial strains but with different effect profiles observed. For example, for the Gram-positive bacterium *S. aureus* ATCC8095, the CIM permeability was significantly increased after treatment with 1 MIC of the GMPE for 4 h (1.20-fold, *p* < 0.05) (Figure 3A). For the Gram-negative bacterium *V. parahaemolyticus* ATCC17802, 1 MIC treatment for only 1 h significantly increased the bacterial CIM permeability by 1.21-fold (*p* < 0.05) (Figure 3E).

The effects of antibacterial components on bacterial cell surface hydrophobicity, membrane fluidity and permeability were also observed for the methanol-phase extract from the edible herbaceous plant *Rumex madaio* Makino in our recent research [14].

### 2.3. Bacterial Cell Morphological Architecture Change Mediated by GMPE Treatment

**Figure 4:**
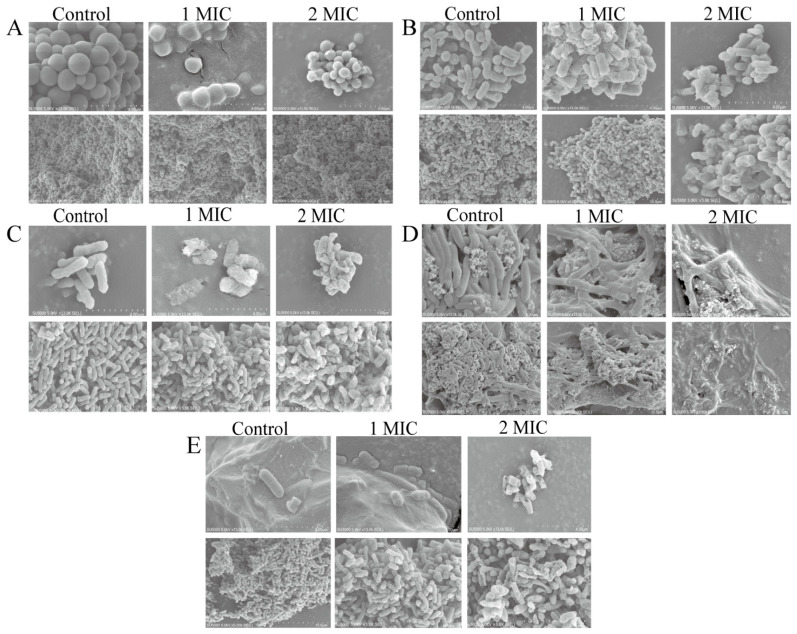
The SEM observation of bacterial cell morphological architecture change induced by the GMPE. (**A**–**E**) *S. aureus* ATCC8095, *A. hydrophila*, *E. sakazakii* CMCC45401, *V. metschnikovii* ATCC700040, and *V. parahaemolyticus* ATCC17802, respectively.

The influence of the GMPE on the cell morphological architecture of *S. aureus* ATCC8095, *A. hydrophila, E. sakazakii* CMCC45401, *V. metschnikovii* ATCC700040 and *V. parahaemolyticus* ATCC17802 was further investigated by scanning electron microscope (SEM) analysis (Figure 4A–E).

As shown in Figure 4A, the control group of Gram-positive *S. aureus* ATCC8095 included spherical cells with an intact, clear and smooth surface. After treatment with 1 MIC of the GMPE for 1 h, *S. aureus* ATCC8095 cells showed an irregular shape with a rough surface. This change was exacerbated with increased concentration of GMPE. The treatment with 2 MIC of the GMPE for 1 h resulted in the disruption of certain *S. aureus* cells.

As shown in Figure 4B–E, for the Gram-negative bacteria tested, the alteration in cell morphological architecture was larger than for the Gram-positive bacteria, which probably resulted from differences in their cell envelope structure [15]. For example, after treatment with 1 MIC of the GMPE for 1 h, some *A. hydrophila* cells were severely damaged (Figure 4B). A similar change was observed for *E. sakazakii* CMCC45401 under the same conditions, which led to conspicuous holes in a rough, wrinkled, and deformed cell surface (Figure 4C). Similarly, compared with the control group with long, rod-shaped, and intact cells, collapsed cell architecture and massive leakage of cell contents were observed for *V. metschnikovii* ATCC700040 after treatment with 2 MIC of the GMPE for 1 h (Figure 4D). The treatment also significantly penetrated the cell membrane structure of *V. parahaemolyticus* ATCC17802 and created large pores in the damaged cells (Figure 4E).

The influence of bioactive compounds from the smaller galangal on cell morphological architecture was also observed by Eumkeb et al. [5]. They isolated galangin, kaempferide and kaempferide-3-O-b-d-glucoside by consecutive extraction with hexane, chloroform and methanol. The combination of amoxicillin and these flavonoids reduced amoxicillin-resistant *Escherichia coli* (AREC) cell numbers. Electron microscopy showed that these combinations damaged the ultrastructure of AREC cells [5].

### 2.4. Differential Transcriptomes Mediated by GMPE Treatment

To obtain insights into gene expression changes mediated by the GMPE at the whole genome level, we further determined the transcriptomes of representative Gram-positive *S. aureus* and Gram-negative *E. sakazakii* pathogens treated by 1 MIC of GMPE for 1 h using the Illumina RNA sequencing technique. *S. aureus* can cause mild skin infections to more severe life-threatening diseases in humans, such as osteomyelitis, pneumonia, and septicemia [16]. *E. sakazakii* is an opportunistic pathogen that has been implicated in infant infections, including bacteremia, infant meningitis, and enterocolitis [17].

The complete lists of differentially expressed genes (DGEs) in *S. aureus* ATCC8095 and *E. sakazakii* CMCC45401 strains were deposited in the National Center for Biotechnology Information (NCBI) SRA database (http://www.ncbi.nlm.nih.gov/sra/ accessed on 3 December 2021) under the accession number PRJNA830289.

#### 2.4.1. The Major Changed Metabolic Pathways in *S. aureus* ATCC8095

**Figure 5:**
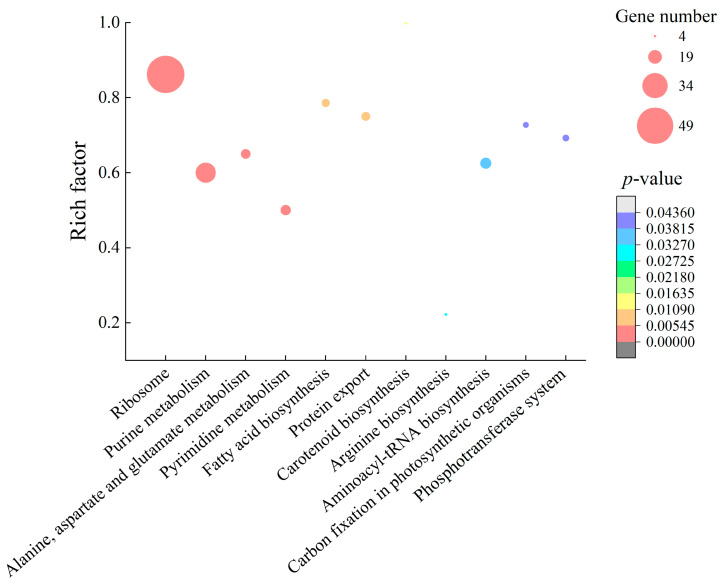
The significantly altered metabolic pathways in *S. aureus* ATCC8095 mediated by the GMPE.

Comparative transcriptomic analyses revealed approximately 36.6% (1007/2751) of *S. aureus* ATCC8095 genes were differently expressed in the treatment group, when compared with the control group. Among these, approximately 676 DGEs showed higher transcriptional levels (fold change, FC ≥ 2.0), while 331 genes were downregulated (FC ≤ 0.5). Approximately 11 significantly altered metabolic pathways were identified against the Kyoto Encyclopedia of Genes and Genomes (KEGG) database, including ribosome biogenesis, purine metabolism, alanine, aspartate and glutamate metabolism, pyrimidine metabolism, fatty acid biosynthesis, protein export, carotenoid biosynthesis, arginine biosynthesis, aminoacyl-tRNA biosynthesis, carbon fixation in photosynthetic organisms, and the phosphotransferase system (PTS) (Figure 5, Table S1).

Approximately 16 DEGs involved in PTS, arginine biosynthesis, and carotenoid biosynthesis were downregulated at the transcription level (0.014- to 0.430-fold, *p* < 0.05) in *S. aureus* ATCC8095 (Table S1). For example, the PTS plays a very important role in carbohydrate transport and controls a variety of cellular process [18,19]. Remarkably, in this study, the expression of seven DEGs encoding sugar, mannose, galactitol, and ascorbate transporter subunits were significantly inhibited (0.021- to 0.430-fold, *p* < 0.05), suggesting inactive transport of these carbohydrates induced by GMPE treatment. Interestingly, in the PTS, two DEGs encoding fructose transporter subunit II C (*EQG65_03560*) and 1-phosphofructokinase (*EQG65_03555*) were highly upregulated by 24.254- and 28.586-fold, respectively. The greatly activated monosaccharide transporter and metabolism were probably more favorable to the bacterium response to the harsh environment induced by the GMPE. In the arginine biosynthesis pathway, the expression of four DEGs encoding key enzymes was also highly repressed (0.014- to 0.094-fold, *p* < 0.05), including arginine deiminase (*EQG65_13875*), ornithine carbamoyl transferase (*EQG65_13870*), ornithine carbamoyl transferase (*EQG65_05940*) and arginase (*EQG65_11195*). Arginine is much more than a common amino acid required for protein synthesis; this basic amino acid also plays an important role in several other aspects of cellular growth and physiology [20]. Additionally, the expression of five DEGs in carotenoid biosynthesis was significantly downregulated (0.103- to 0.312- fold, *p* < 0.05).

Comparative transcriptomic analyses also revealed some upregulated DEGs involved in nine significantly enhanced metabolic pathways in *S. aureus* ATCC8095 (*p* < 0.05). GMPE treatment triggered significant changes in nucleotide metabolism, including 34 upregulated DEGs (2.344- to 30.276-fold, *p* < 0.05) in purine and pyrimidine metabolism. For example, the DEG encoding the aspartate carbamoyltransferase was highly upregulated (*EQG65_06115*, 30.276-fold). It catalyzes the first committed step in de novo pyrimidine biosynthesis in bacteria, and the acquisition of nucleotides is a vital process in all living cells [21]. The DEGs encoding large and small subunits (*EQG65_06130*, *EQG65_06125*) of carbamoyl-phosphate synthetase, which is a key enzyme in both pyrimidine and arginine biosynthesis [22], were also highly enhanced (15.489, and 19.096-fold, respectively). Additionally, adenylosuccinate synthase (*EQG65_00095*, 16.494-fold) plays an important role in the salvage pathway in purine nucleotide biosynthesis [23].

In alanine, aspartate and glutamate metabolism, the expression of six DEGs was also significantly upregulated at the transcription level (2.208- to 19.096-fold, *p* < 0.05) (Table S1). Additionally, approximately 15 DGEs in the aminoacyl-tRNA biosynthesis pathway were significantly upregulated (2.034- to 4.401-fold, *p* < 0.05).

Ribosomes are macromolecular complexes for cellular protein synthesis. The biogenesis of ribosomes is an intricate multistep process that involves the transcription of ribosomal DNA (rDNA), the processing of ribosomal RNA (rRNA), and the assembly of rRNA with ribosomal proteins to form active ribosomes [24]. In this study, the DEGs (*n* = 50) linked to ribosome biogenesis were all significantly upregulated (2.25- to 8.05-fold, *p* < 0.05). Meanwhile, in protein export, the expression of all the DEGs (except *EQG65_13950*) were significantly enhanced (2.006- to 3.472-fold, *p* < 0.05), such as the cooperation of signal peptidase IB (*EQG65_04850*), protein translocase subunit SecDF (*EQG65_08470*), lipoprotein signal peptidase (*EQG65_06095*) and signal peptidase (*EQG65_04845*), which are crucial in the stabilization of protein-folding intermediates, protein assembly and disassembly, and protein secretion and degradation, particularly in harsh environments [25].

The upregulated nucleotide and amino acid metabolism, protein synthesis and export in *S. aureus* ATCC8095 may have contributed to resistance to the external adverse environment caused by the GMPE in order to maintain bacterial cell structure and functional stability.

#### 2.4.2. The Major Altered Metabolic Pathways in *E. sakazakii* CMCC45401

**Figure 6:**
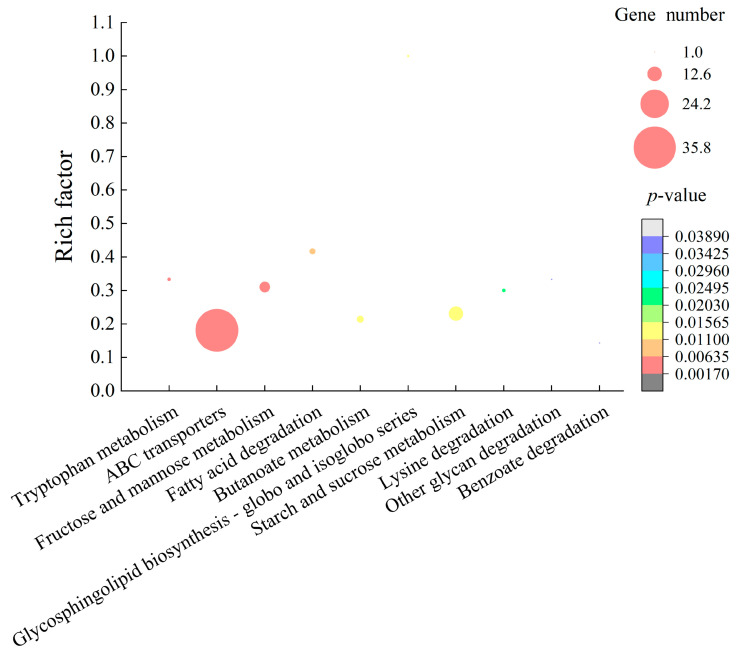
The significantly altered metabolic pathways in *E. sakazakii* CMCC45401 mediated by the GMPE.

For the Gram-negative *E. sakazakii* CMCC45401, approximately 11.2% (432/3841) of the bacterial genes were expressed differently at the transcription level in the treatment group, when compared with the control group. Among these, approximately 214 DEGs were significantly upregulated, whereas 218 showed lower transcription levels (*p* < 0.05). Gene set enrichment analysis (GSEA) of these GEGs against the KEGG database revealed ten significantly changed metabolic pathways in *E. sakazakii* CMCC45401, including pathways involved in tryptophan metabolism, ABC transporters, fructose and mannose metabolism, fatty acid degradation, butanoate metabolism, glycosphingolipid biosynthesis, starch and sucrose metabolism, lysine degradation, other glycan degradation, and benzoate degradation (Figure 6, Table S2).

Comparative transcriptomic analyses revealed four significantly enhanced degradation pathways induced by the GMPE, including fatty acid degradation, lysine degradation, other glycan degradation, and benzoate degradation in *E. sakazakii* CMCC45401. For example, three DEGs were significantly upregulated in the lysine degradation pathway (1.807- to 2.441-fold, *p* < 0.05), such as *AFK63_16660* (2.441-fold) encoding succinate-semialdehyde dehydrogenase. This key enzyme contributes to many metabolic pathways, including the tricarboxylic acid cycle (TCA) [26]. Meanwhile, the expression of approximately five DEGs was significantly enhanced in fatty acid degradation (1.935- to 5.512-fold, *p* < 0.05), which encoded the long-chain fatty acid-CoA ligase (*AFK63_06955*), 3-ketoacyl-CoA thiolase (*AFK63_17080*), acyl-CoA dehydrogenase (*AFK63_14360*), multifunctional fatty acid oxidation complex subunit alpha (*AFK63_17075*), and alcohol dehydrogenase (*AFK63_08175*). These upregulated DEGs implied inactive protein and cell membrane synthesis in *E. sakazakii* CMCC45401.

In starch and sucrose metabolism pathways, approximately 12 DEGs were significantly upregulated (1.901- to 7.040-fold, *p* < 0.05). In addition, the expression of nine DEGs involved in fructose and mannose metabolism was significantly enhanced at the transcription level (1.769- to 13.341-fold, *p* < 0.05). Notably, the gene encoding mannose-1-phosphate guanyltransferase (*AFK63_05420*), which is involved in the maintenance of cell wall integrity and/or glycosylation [27], was highly expressed (13.341-fold, *p* < 0.05).

In the tryptophan metabolism pathway, three DEGs were significantly upregulated (2.043- to 3.317-fold, *p* < 0.05) in *E. sakazakii* CMCC45401. For example, the expression of the DEG encoding hydroperoxidase (*AFK63_10145*) was significantly increased by 2.043-fold. Alfonso-Prieto et al. reported that hydroperoxidase efficiently degraded hydrogen peroxide into water and oxygen to prevent oxidative damage to cells (Alfonso-Prieto et al. 2009). Moreover, the DEG encoding peroxidase (*AFK63_02085*) was also upregulated at the transcription level (*AFK63_02085,* 3.317-fold, *p* < 0.05).

ABC transporter proteins utilize the energy stored in adenosine triphosphate (ATP) to transport various substrates across the bacterial cell membrane [28]. They mediate either the uptake of essential nutrients into the cell or the export of lipids, metabolites, and other small molecules out of the cell [29]. In this study, comparative transcriptomic analyses revealed approximately 36 DEGs encoding ABC transporter proteins induced by the GMPE. Among these, the expression of 21 DEGs, which mainly transport sugar, ribose, maltose, and phosphate, was significantly upregulated at the transcription level (1.779- to 3.497-fold), whereas 15 DEGs, which mainly transport amino acids, were significantly downregulated (0.204-to 0.435-fold) (*p* < 0.05), consistent with the above significantly altered metabolic pathways in *E. sakazakii* CMCC45401 (Table S2).

Taken together, the obtained distinct transcriptomic profiles indicated different antibacterial action modes of the GMPE against the Gram-positive *S. aureus* ATCC8095 and Gram-negative *E. sakazakii* CMCC45401. Additionally, real-time reverse transcription polymerase chain reaction (RT-PCR) assay was performed to test eight representative DEGs, and the results were generally consistent with the transcriptome analyses (data not shown).

### 2.5. Separation of Antibacterial Components in the GMPE

**Figure 7:**
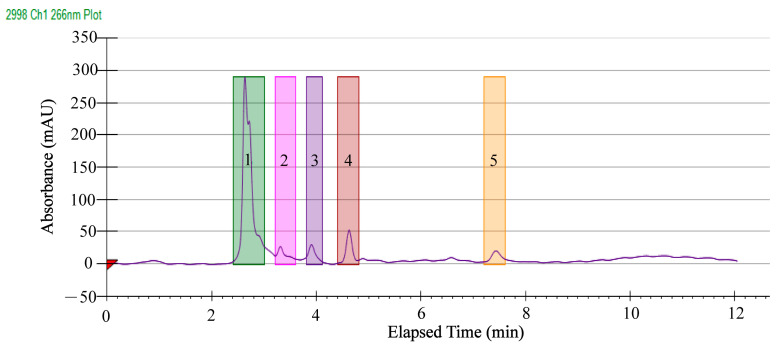
The Prep-HPLC diagram of purifying the GMPE.

In order to identify antibacterial components in the rhizomes of *A. officinarum* Hance, the GMPE was further separated by HPLC analysis. As shown in Figure 7, five major clear peaks were observed, which were eluted from 2.4 to 7.8 min using a gradient elution program, including Fraction 1 (2.40 min), Fraction 2 (3.19 min), Fraction 3 (3.82 min), Fraction 4 (4.43 min), and Fraction 5 (7.03 min). Notably, Fraction 1 contained the most abundant compounds compared to the others. The absorbance values at 266 nm were increased when higher concentrations of the GMPE were applied (figures not shown). These results indicated that a good resolution was achieved for the separation of antibacterial components in the GMPE.

Using the same conditions, Prep-HPLC was carried out and a number of components in the five major fractions were collected and concentrated to verify their antibacterial activity. The observed antibacterial effect of each single fraction was not as strong as the GMPE, which could be explained by a synergistic effect. A synergistic effect indicates that a combination of several compounds in the plant results in stronger activity than the individual active compound for that activity [30]. In this study, the results showed that the DIZ values of the Fraction 1 were significantly larger than for the control groups against *S. aureus* ATCC8095 (10.8 mm), and *E. sakazakii* CMCC45401 (11.3 mm). Similar results were observed for Fraction 4, whereas the other three fractions showed no significant activity (data not shown). These results indicated that antibacterial components existed in Fractions 1, and 4 of the GMPE; therefore, these two fractions were subjected to identification of potential antibacterial compounds in this plant as described in the following analysis.

### 2.6. Identification of Potential Antibacterial Compounds in the GMPE

Based on the above results, the major antibacterial compounds in Fraction 1 and Fraction 4 of the GMPE were identified using UHPLC-MS techniques. As shown in Table 3 and Table 4, the UHPLC-MS analysis revealed 46 and 45 major components in Fraction 1 and Fraction 4, respectively.

Remarkably, o-methoxy cinnamaldehyde was the most abundant compound in Fraction 1 of the GMPE (40.12%), followed by phosphoric acid (6.90%), indole (2.30%), acetamide (2.20%), L-pipecolic acid (1.95%), 12,13-DiHOME (1.91%), kojibiose (1.73%), β-D-fructose 2-phosphate (1.73%), and L-asparagine (1.64%). The o-methoxy cinnamaldehyde was classified into phenols, which can disrupt bacterial cell membranes, prevent biofilm formation, and inhibit bacterial motility [31]. Bactericidal activity of phosphoric acid against *Enterococcus faecalis* has been reported [32]. Alkaloids (indole, and acetamide) are plant secondary metabolites, which have been shown to have potent pharmacological activity [33]. The other 38 identified compounds accounted for 0.92% to 0.01% of Fraction 1 of the GMPE (Table 3).

In Fraction 4 of the GMPE, notably, p-octopamine was the major potential antibacterial compound (62.64%), which was also classified into phenols. The acetamide was the second most abundant component in Fraction 4 (14.30%), followed by indole (4.90%), 12,13-DiHOME (2.85%), phosphoric acid (2.64%), 3α,6β-ditigloyloxytropan-7β-ol (1.71%), sarracine (1.71%), lubiprostone (1.36%), and o-methoxycinnamaldehyde (1.35%). These components accounted for 93.46% of Fraction 4, while the remainder was composed of the other 36 compounds (Table 4). Zhang et al. reported that organic acids showed good antioxidant activity and inhibition against *E. coli*, *S. aureus*, and *Bacillus subtilis* [34]. Previous research has also indicated that terpenoid compounds had a broad antibacterial activity against Gram-positive and Gram-negative bacteria, which suggests that they could be employed as a potential source of new natural products with effective medicinal properties [35].

The overuse or misuse of antibiotics drives the evolution of resistance of pathogenic bacteria, which results in increased mortality, hospitalization, and healthcare costs [36]. In this study, the identified antibacterial ingredients in the GMPE could help meet the increased demand for the use of natural antibacterial compounds from pharmacophagous plants, such as extracts of herbs and spices [37].

## 3. Materials and Methods

### 3.1. Plant Samples and Bioactive Ingredient Extraction

The rhizomes of *A. officinarum* Hance are edible and widely used as food spices having a special spicy smell. They are cylindrical, mostly curved, and branched and are of 5 to 9 cm in length and 1 to 1.5 cm in diameter. Their surface is dark brown with fine longitudinal wrinkles and gray-brown wavy links (Pharmacopoeia of the Peoples’ Republic of China, 2020 Edition). A quantity of 1000 g of dried rhizomes samples of *A. officinarum* Hance were collected from the Chuxiong Yi Autonomous Prefecture in Yunnan Province, China in April 2021. Bioactive ingredients of the samples were extracted using the CME method as previously described [14,38] with minor modification. Briefly, the dried rhizome samples of *A. officinarum* Hance were crushed using an FW-135 high-speed crusher (Beijing Kangtuo Medical Instruments Co., Ltd., Beijing, China). Then, 10.0 g of the powder was mixed with 99-mL chloroform: methanol (2:1, *v*/*v*) at a solid to liquid ratio of 1:10 (*m*/*v*) for 5 h. A quantity of 60 mL of H_2_O (Analytical grade, Merck KGaA, Darmstadt, Germany) was then added, and fully mixed. The mixture was filtered through a 20–25 µm membrane (Shanghai Sangon Biological Engineeing Technology and Service Co., Ltd., Shanghai, China). The extraction was performed twice, and the methanol phase was separated from the chloroform phase with a separatory funnel. The methanol-phase filtrate was concentrated using an ALPHA 2–4 LD Plus Freeze Dryer (Martin Christ, Osterode, Germany) at −80 °C for 48 h. The chloroform-phase filtrate was evaporated and concentrated on pasting using a Rotary Evaporator (IKA, Staufen, Germany). The solid residue was dissolved with an appropriate solvent and stored in a refrigerator (4 °C) until used for analysis. The chloroform and methanol (Analytical Grade) were purchased from Collins (Shanghai, China).

### 3.2. Antibacterial Activity Assays

The antibacterial activity of the extracts was measured using a disc diffusion method issued by the Clinical and Laboratory Standards Institute (CLSI) (2018, CLSI, M100-S23) using Mueller–Hinton (M-H) agar (CM337) and Mueller–Hinton broth (M391) (OXOID, Basingstoke, UK). Briefly, 10 µL of crude extract (500 µg/mL) was added onto each sterile disk (Whatman No. 5, 6 mm diameter) on M–H agar plates. A gentamicin disc (10 µg, OXOID, Basingstoke, UK) was used as a positive control, while the methanol-phase, with sterile ultrapure water and chloroform-phase with ethanol, was used as a negative control [14]. The plates were incubated at 37 °C for 16–18 h. All experiments were performed independently in triplicate. The diameters of the bacteriostatic circles were measured and calculated [14]. The antibacterial activity of the disc diffusion method was defined where the DIZ values were significantly different from negative controls.

Broth dilution testing (microdilution) (2018, CLSI, M100-S18) was used to determine the MICs of the extracts [14]. A concentration of 1 MIC was the lowest concentration of the test substances that prevented visible growth of the microorganisms, while 2 MIC was double 1 MIC [39]. The MBC was defined as the lowest concentration of an agent that produced no growth of subculture [40]. The MBCs of the extracts were examined by subculturing 100 μL/well bacterial culture from the MIC assay onto fresh M–H agar plates. The lowest concentration of the samples, which showed no bacterial growth after this subculturing, was recorded as the MBC, indicating that the bacterial cells were completely killed [40]. The bacterial strains, culture media, and incubation conditions used in this study were the same as described in our previous research [14].

### 3.3. Bacterial Cell Structure Assays

Bacterial cell surface hydrophobicity, membrane fluidity and CIM permeability were determined using the same methods and chemical regents as described in our previous studies [14]. Each of the bacterial strains at the mid-logarithmic growth phase (LGP) was collected, washed, and treated using 1 MIC and 2 MIC of the GMPE at 37 °C for 1 h in the cell surface hydrophobicity and membrane fluidity assays, and for 4 h in the CIM permeability assay.

The hydrophobicity assay was performed as previously described by Yan et al. [41] with minor modification. Briefly, 1 mL of bacterial suspension (OD_600nm_ values of 0.5) was mixed with an equal volume of *n*-hexadecane (China National Pharmaceutical Group Corporation Co., Ltd., Shanghai, China), rotated for 1 min and then stood at room temperature for 30 min. The absorbance of the aqueous phase was measured at OD_600 nm_ using a BioTek Synergy 2 multi-mode plate reader (BioTek, Burlington, VT, USA). The percentage of hydrophobicity was expressed as hydrophobicity% = [(*A*_0_ − *A*)/*A*_0_] × 100, where *A*_0_ and *A* are the absorbance values of the aqueous phase before and after contact with *n*-hexadecane.

To measure the membrane fluidity, a 200 µL/well of bacterial suspension was mixed with 2 µL of 10 mM 1,6-diphenyl-1,3,5-hexatriene (DPH) (Sangon, China). The fluorimeter’s vertically polarized light was 360 nm and the emitted light was measured at 460 nm vertically (Ivv) and horizontally (Ivh) through a polarizer compared to the excited light using a BioTek Synergy 2 multi-mode plate reader (BioTek, Burlington, VT, USA). The grating factor (G) used was 0.85. The membrane fluidity (rDPH) was calculated according to the following formula: (Ivv-G × Ivh)/Ivv + 2 × G × Ivh, and G = 0.85 [42].

For the CIM permeability assay [43], briefly, the treated bacterial suspension was centrifuged, washed, and resuspended in 0.1 M PBS solution (Shanghai Sangon Biological Engineering Technology Services Co., Ltd. (Shanghai, China) to adjust the absorbance at a 600 nm value of 0.4. Then, 200 μL/well of the cell suspension was added into a sterile 96-well plate, and 2.5 μL/well of 10 mM (O-nitrophenyl-β-D galactopyranoside, ONPG) was added. The absorbance values at OD_415nm_ of each well were determined using a BioTek Synergy2 multi-mode plate reader (BioTek, Burlington, VT, USA) at 37 °C for every 30 min for 4 h. The OD_415nm_ values from the treatment groups were defined as OD1, while the untreated control was defined as OD2.

The bacterial cultures treated with 1 MIC and 2 MIC of the GMPE at 37 °C for 1 h were also collected for SEM observation using thermal field emission SEM (Hitachi, SU5000, Tokyo, Japan) with accelerating voltages of 5–10 kV. The bacterial cells were washed, fixed, dehydrated, dried, and gold-covered by cathodic spraying according to the method described previously [44].

### 3.4. Prep-HPLC Analysis

The GMPE was separated by Prep-HPLC using a Waters 2707 autosampler (Waters, Milford, MA, USA) linked with a UPLC Sunfifire C18 column (5 µm, 10 *×* 250 mm) (Waters, Milford, MA, USA) as described in our previous research [14] with minor modification. Briefly, aliquots (10 mg/mL) of the GMPE sample were resolved in ultrapure water (Analytical grade, Merck KGaA, Darmstadt, Germany) and centrifuged at 8000 rpm for 30 min at 4 °C. The supernatant was filtered through a 0.22 µm membrane (Shanghai Titan Technology Co., Ltd., Shanghai, China). The filtrate was used for further analysis. Prep-HPLC was run at the following parameters: column temperature, 40 °C; injection volume, 100 µL; and mobile phase of 90% methanol (eluent A) and water (eluent B) at a flow rate of 4 mL/min (isocratic elution: 0–15 min, 20% eluent A and 80% eluent B). Photo-diode array (PDA) spectra were measured at wavelengths ranging from 200 to 600 nm.

### 3.5. HPLC-MS Analysis

The HPLC-MS analysis was carried out by Shanghai Hoogen Biotech (Shanghai, China) using the EXIONLC System (Sciex, Framingham, MA, USA) linked with a liquid chromatographic column (1.8 μm, 2.1 × 100 mm) (Waters, Milford, MA, USA). The mobile phase A contained 0.1% formic acid in H_2_O (*v*/*v*), and the mobile phase B was acetonitrile (Merck KGaA, Darmstadt, Germany); column temperature: 40 °C; auto-sampler temperature: 4 °C; and injection volume: 2 µL. Typical ion source parameters were: IonSpray voltage: +5500/−4500 V; curtain gas: 35 psi; temperature: 400 °C; ion source Gas 1:60 psi; ion source Gas 2: 60 psi; and declustering potential (DP): ±100 V. All mass spectrometry data acquisition and quantitative analysis of target compounds were performed using SCIEX Analyst WorkStation Software (Version 1.6.3, Hoogen Biotech, Shanghai, China). An in-house R program and database were used for fraction detection and annotation (Shanghai Hoogen Biotech, Shanghai, China) [14].

### 3.6. Illumina RNA Sequencing

The bacteria strains at mid-LGP treated with 1 MIC of the GMPE at 37 °C for 1 h were individually collected for the transcriptome analysis. Total RNA extraction and DNA removal were performed using an RNeasy Protect Bacteria Mini Kit (QIAGEN Biotech Co. Ltd., Dusseldorf, Germany), a QIAGEN RNeasy Mini Kit (QIAGEN), and an RNase-Free DNase Set (QIAGEN), according to the manufacturer’s protocols. Three independently prepared RNA samples were used in each Illumina RNA-sequencing experiment. The sequencing library construction and Illumina sequencing were conducted at Shanghai Majorbio Bio-pharm Technology Co. Ltd., China using an Illumina HiSeq 4000 SBS Kit platform as described previously [45]. Only high quality reads that passed the Illumina quality filters were used for sequence analyses [45].

Bacterial cell cultures grown to the mid-LGP were harvested by centrifugation at 8000 g for 10 min at 4 °C. The supernatant was removed, and bacterial cell pellets were used for the total RNA extraction, and reverse transcription reactions using an RNeasy Mini Kit (Qiagen, Germany) and a PrimeScript™ RT reagent Kit with a gDNA Eraser (Perfect Real Time) (TaKaRa, Kusatsu, Japan) kit, according to the manufacturers’ instructions. Relative quantitative PCR reactions were performed with a TB Green^®^ Premix Ex Taq™ II (Tli RNaseH Plus) (TaKaRa, Kusatsu, Japan) kit using a 7500 Fast Real-Time PCR Instrument (Applied Biosystems, USA) [46]. The 16S rRNA was used as the internal reference gene, and the 2^−ΔΔCt^ method was used to calculate the relative expression between the target and the internal reference genes [47]. The primers were synthesized by Sangon (Shanghai, China). All tests were performed in triplicate.

### 3.7. Statistical Analysis

Expression of each gene was calculated using RNA-Seq by expectation-maximization (http://deweylab.github.io/RSEM/, accessed on 3 December 2021) [14,46]. Genes with the criteria, fold-changes ≥2.0 or ≤0.5, and *p*-values < 0.05, relative to the control, were defined as DEGs. These DEGs were used for the GSEA against the KEGG database (https://www.genome.jp/kegg/, accessed on 3 December 2021). The data were analyzed using SPSS Statistics software (version 22, IBM, Armonk, NY, USA).

## 4. Conclusions

This study was the first to use a chloroform and methanol extraction method to investigate antibacterial activity and components of the rhizomes of *A. officinarum* Hance. The results showed that the growth of four species of Gram-negative, and one species of Gram-positive, pathogenic bacteria were significantly inhibited by the GMPE (*p* < 0.05). The observed MIC values against *A. hydrophila*, *E. sakazakii* CMCC45401, *S. aureus* ATCC8095, *V. metschnikovii* ATCC700040, and *V. parahaemolyticus* ATCC17802 were 1.95 mg/mL, 3.9 mg/mL, 3.9 mg/mL, 7.81 mg/mL, and 3.90 mg/mL, respectively. The GMPE treatment changed the bacterial cell surface hydrophobicity, membrane fluidity and/or permeability, showing different effect profiles between Gram-negative and Gram-positive pathogens. Comparative transcriptomic analyses revealed approximately eleven and ten significantly altered metabolic pathways in representative Gram-positive *Staphylococcus aureus* and Gram-negative *Enterobacter sakazakii* pathogens, respectively (*p* < 0.05), demonstrating different antibacterial action modes of the GMPE. The GMPE was further separated using a Prep-HPLC technique, and approximately 46 and 45 different compounds in two major component fractions (Fractions 1 and 4) were identified using UHPLC-MS techniques. o-Methoxy cinnamaldehyde (40.12%) and p-octopamine (62.64%) were the most abundant compounds in Fractions 1 and 4, respectively. The results of this study help to address the increased need to develop natural products from pharmacophagous plants against common pathogenic bacteria.

## Figures and Tables

**Table 1:** Antimicrobial activity of the GMPE and GCPE.

Bacterial Strain	DIZ (Diameter, mm)	
	GMPE	GCPE
*Aeromonas hydrophila*	16.03 ± 0.71 ^a^	12.03 ± 0.01 ^a^
*Aeromonas hydrophila* ATCC35654	—	—
*Enterobacter sakazakii* CMCC45401	11.54 ± 0.71 ^a^	—
*Escherichia coli* K12	—	10.5 ± 0.71 ^a^
*Enterobacter cloacae* ATCC13047	—	—
*Escherichia coli* ATCC8739	—	—
*Escherichia coli* ATCC25922	—	—
*Enterobacter cloacae*	—	—
*Listeria monocytogenes* ATCC19115	—	7.25 ± 0.35 ^a^
*Pseudomonas aeruginosa* ATCC9027	—	—
*Pseudomonas aeruginosa* ATCC27853	—	—
*Staphylococcus aureus* GIM1.160	—	12.75 ± 1.06 ^a^
*Staphylococcus aureus* ATCC8095	12.32 ± 0.35 ^a^	—
*Staphylococcus aureus* ATCC29213	12.00 ± 1.41 ^a^	—
*Staphylococcus aureus* GIM1.441	12.52 ± 0.71 ^a^	—
*Staphylococcus aureus* ATCC25923	—	—
*Staphylococcus aureus* ATCC6538	—	—
*Salmonella paratyphi-A* CMCC50093	—	—
*Salmonella enterica* subsp. *enterica* (*ex* Kauffmann and Edwards) Le Minor and Popoff serovar Choleraesuis ATCC13312	—	—
*Salmonellaenterica* subsp. *enterica* (*ex* Kauffmann and Edwards) Le Minor and Popoff serovar Vellore ATCC15611	—	8.50 ± 0.05 ^a^
*Shigella dysenteriae* CMCC51252	—	—
*Shigella flexneri* CMCC51572	—	—
*Shigella flexneri* ATCC12022	—	—
*Shigella flexneri* ATCC51574	—	—
*Shigella sonnei* ATCC25931	—	—
*Shigella sonnei* CMCC51592	—	—
*Vibrio alginolyticus* ATCC17749	—	—
*Vibrio alginolyticus* ATCC33787	—	—
*Vibrio alginolyticus*	—	—
*Vibrio harvey* ATCC BAA-1117	—	—
*Vibrio harveyi* ATCC33842	—	—
*Vibrio parahaemolyticus* B3-13	—	—
*Vibrio parahaemolyticus* B4-10	—	—
*Vibrio parahaemolyticus* B5-29	—	—
*Vibrio parahaemolyticus* B9-35	—	—
*Vibrio parahaemolyticus* ATCC17802	11.03 ± 1.40 ^a^	—
*Vibrio vulnificus* ATCC27562	—	—
*Vibrio vulnificus*	—	7.75 ± 0.35 ^a^
*Vibrio fluvialis* ATCC33809	—	7.02 ± 0.01 ^a^
*Vibrio metschnikovii* ATCC700040	9.05 ± 0.01 ^a^	11.25 ± 0.35 ^a^
*Vibrio mimicus* bio-56759	—	8.25 ± 0.35 ^a^

Note: values are expressed as mean ± S.D. of three parallel measurements; a: significant difference compared with sterile ultrapure water and ethanol groups for the GMPE and GCPE groups, respectively (*p* < 0.05); —: no antibacterial activity. DIZ includes the disk diameter (6 mm).

**Table 2:** The MIC and MBC values of the GMPE against the five species of bacteria.

Bacterial Strain	MIC (mg/mL)	MBC (mg/mL)
*A. hydrophila*	1.95	3.90
*E. sakazakii* CMCC45401	3.90	7.81
*S. aureus* ATCC8095	3.90	7.81
*V. metschnikovii* ATCC700040	7.81	15.62
*V. parahaemolyticus* ATCC17802	3.90	7.81

**Table 3:** Identification of potential antibacterial ingredients in Fraction 1 of the GMPE.

No	Compound	Classification	RT (min)	Formula	Peak Area (%)
1	o-Methoxy cinnamaldehyde	Phenols	11.6	C_10_H_10_O_2_	40.12
2	Phosphoric acid	Organic acids	0.65	H_3_O_4_P	6.90
3	Indole	Alkaloids	3.82	C_8_H_7_N	2.30
4	Acetamide	Alkaloids	13.95	C_2_H_5_NO	2.20
5	L-Pipecolic acid	Amino acid and derivatives	1.47	C_6_H_11_NO_2_	1.95
6	12,13-DHOME	Fatty acyls	11.88	C_18_H_34_O_4_	1.91
7	Kojibiose	Fatty acyls	0.72	C_12_H_22_O_11_	1.73
8	β-D-Fructose 2-phosphate	Organooxygen compounds	0.75	C_6_H_13_O_9_P	1.73
9	L-Asparagine	Amino acids and derivatives	0.64	C_4_H_8_N_2_O_3_	1.64
10	3α,6β-Ditigloyloxytropan-7β-ol	Alkaloids	13.21	C_18_H_27_NO_5_	0.92
11	D-α-Aminobutyric acid	Amino acids and derivatives	0.65	C_4_H_9_NO_2_	0.81
12	Proline; L-Proline	Amino acids and derivatives	0.73	C_5_H_9_NO_2_	0.66
13	D-Proline	Amino acids and derivatives	0.76	C_5_H_9_NO_2_	0.66
14	L-Aspartic acid	Amino acids and derivatives	0.63	C_4_H_7_NO_4_	0.64
15	Maltol	Phenols	0.9	C_6_H_6_O_3_	0.54
16	cis-Aconitic acid	Organic acids and derivatives	1.46	C_6_H_6_O_6_	0.54
17	L-Glutamic acid	Amino acids and derivatives	0.66	C_5_H_9_NO_4_	0.47
18	DL-Alanine; L-Alanine	Amino acids and derivatives	0.64	C_3_H_7_NO_2_	0.39
19	Epicatechin; (+)-Epicatechin	Flavonoids	5.08	C_15_H_14_O_6_	0.38
20	L-Ornithine	Amino acids and derivatives	0.55	C_5_H_12_N_2_O_2_	0.35
21	L-Arginine	Amino acids and derivatives	0.6	C_6_H_14_N_4_O_2_	0.35
22	Sucrose	Carbohydrates	0.89	C_12_H_22_O_11_	0.35
23	Erucic acid	Fatty acyls	13.28	C_22_H_42_O_2_	0.31
24	O-Acetyl ethanolamine	Alkaloids	0.67	C_4_H_9_NO_2_	0.31
25	Linamarin	Organooxygen compounds	0.71	C_10_H_17_NO_6_	0.30
26	Ethyl caproate	Esters	0.74	C_8_H_16_O_2_	0.29
27	Lubiprostone	Fatty acyls	12.75	C_20_H_32_F_2_O_5_	0.28
28	Trimethoprim	Pyrimidines	5.08	C_14_H_18_N_4_O_3_	0.25
29	L-Pipecolic acid	Amino acids and derivatives	0.69	C_6_H_11_NO_2_	0.23
30	Pyrrolidonecarboxylic acid	Carboxylic acids and derivatives	0.67	C_5_H_7_NO_3_	0.23
31	L-Carnitine	Vitamins	0.69	C_7_H_15_NO_3_	0.23
32	Phosphorylcholine	Choline	0.67	C_5_H_14_NO_4_P	0.22
33	8,9-DiHETrE	Fatty acyls	13.03	C_20_H_34_O_4_	0.21
34	Procyanidin B2	Flavonoids	4.78	C_30_H_26_O_12_	0.20
35	2-Picolinic acid	Organic acids	1.33	C_6_H_5_NO_2_	0.19
36	8-Geranyloxypsoralen	Coumarins	13.29	C_21_H_22_O_4_	0.17
37	Alpha-D-Glucose; D-Tagatose	Carbohydrates; organooxygen compounds	0.76	C_6_H_12_O_6_	0.17
38	Safrole	Benzodioxols	12.26	C_10_H_10_O_2_	0.12
39	Thiamine	Vitamins	0.70	C_12_H_16_N_4_OS	0.12
40	Caryophyllene oxide	Sesquiterpenes	11.66	C_15_H_24_O	0.11
41	α-Tocopherol	Phenols	13.37	C_29_H_50_O_2_	0.11
42	L-Lysine	Amino acids and derivatives	0.64	C_6_H_14_N_2_O_2_	0.11
43	Sarracine	Alkaloids	13.14	C_18_H_27_NO_5_	0.08
44	Palmitoylethanolamide	Fatty acid amides	12.61	C1_8_H_37_NO_2_	0.08
45	2-Hydroxyethanesulfonate	Organic acids	0.76	C_2_H_6_O_4_S	0.05
46	Demethoxyencecalin	Phenols	11.80	C_13_H_14_O_2_	0.01

**Table 4:** Identification of potential antibacterial ingredients in Fraction 4 of the GMPE.

No	Compound	Classification	RT (min)	Formula	Peak Area (%)
1	p-Octopamine	Phenols	3.84	C_8_H_11_NO_2_	62.64
2	Acetamide	Alkaloids	13.95	C_2_H_5_NO	14.30
3	Indole	Alkaloids	3.82	C_8_H_7_N	4.90
4	12,13-DiHOME	Fatty acyls	11.88	C_18_H_34_O_4_	2.85
5	Phosphoric acid	Organic acids	0.65	H_3_O_4_P	2.64
6	3α,6β-ditigloyloxytropan-7β-ol	Alkaloids	13.21	C_18_H_27_NO_5_	1.71
7	Sarracine	Alkaloids	13.14	C_18_H_27_NO_5_	1.71
8	Lubiprostone	Fatty acyls	12.75	C_20_H_32_F_2_O_5_	1.36
9	o-Methoxycinnamaldehyde	Phenols	11.6	C_10_H_10_O_2_	1.35
10	Epicatechin; (+)-epicatechin	Flavonoids	5.08	C_15_H_14_O_6_	0.85
11	Erucic acid	Fatty acyls	13.28	C_22_H_42_O_2_	0.75
12	Trimethoprim	Pyrimidines	5.08	C_14_H_18_N_4_O_3_	0.64
13	8,9-DiHETrE	Fatty acyls	13.03	C_20_H_34_O_4_	0.46
14	8-Geranyloxypsoralen	Coumarins	13.29	C_21_H_22_O_4_	0.42
15	4-Hydroxyphenylacetylglutamic acid	Others	12.99	C_13_H_15_NO_6_	0.35
16	L-Pipecolic acid; pipecolic acid; (2E)-decanoyl-ACP	Amino acids and derivatives; Carboxylic acids and derivatives	1.47	C_6_H_11_NO_2_	0.34
17	D-α-aminobutyric acid	Carboxylic acids and derivatives	0.65	C_4_H_9_NO_2_	0.31
18	Uracil	Nucleotides and its derivates	1.91	C_4_H_4_N_2_O_2_	0.31
19	Caryophyllene oxide	Sesquiterpenes	11.66	C_15_H_24_O	0.27
20	L-epicatechin	Flavonoids	5.08	C_15_H_14_O_6_	0.26
21	Palmitoylethanolamide	Fatty acid amides	12.61	C_18_H_37_NO_2_	0.21
22	Safrole	Benzodioxoles	12.26	C_10_H_10_O_2_	0.18
23	Oleic acid; vaccenic acid; petroselinic acid	Fatty acyls	13.03	C_18_H_34_O_2_	0.18
24	Aristolindiquinone	Quinones	11.14	C_12_H_10_O_4_	0.18
25	Cholesterol	Steroids and steroid derivatives	11.86	C_27_H_46_O	0.16
26	Cinchonine	Alkaloids	11.99	C_19_H_22_N_2_O	0.15
27	L-glutamic acid	Amino acids and derivatives	0.66	C_5_H_9_NO_4_	0.15
28	L-threonine	Amino acids and derivatives	0.64	C_4_H_9_NO_3_	0.15
29	L-homoserine	Amino acid and derivatives	0.67	C_4_H_9_NO_3_	0.13
30	AICAR	Imidazole ribonucleosides and ribonucleotides	13.28	C_9_H_15_N_4_O_8_P	0.13
31	α-cyperone	Sesquiterpenoids	12.2	C_15_H_22_O	0.13
32	Vidarabine	Purine nucleosides	2.28	C_10_H_13_N_5_O_4_	0.13
33	Procyanidin B2	Flavonoids	4.78	C_30_H_26_O_12_	0.12
34	Valerenic acid	Sesquiterpenoids	11.24	C_15_H_22_O_2_	0.12
35	L-asparagine	Amino acids and derivatives	0.64	C_4_H_8_N_2_O_3_	0.12
36	Proline; L-proline	Amino acids and derivatives	0.73	C_5_H_9_NO_2_	0.12
37	D-proline	Carboxylic acids and derivatives	0.76	C_5_H_9_NO_2_	0.12
38	Bisabolol oxide A	Sesquiterpenoids	11.5	C_15_H_26_O_2_	0.11
39	β-Sitosterol; β-sitosterol	Steroids and steroid derivatives	12.93	C_29_H_50_O	0.10
40	Kirenol	Diterpenoids	13.16	C_20_H_34_O_4_	0.10
41	Trans-caryophyllene	Sesquiterpenes	12.12	C_15_H_24_	0.09
42	Styrene oxide	Benzene and substituted derivatives	5.94	C_8_H_8_O	0.09
43	Levamisole	Imidazothiazoles	12.04	C_11_H_12_N_2_S	0.08
44	Betulin	Triterpenoids	12.32	C_30_H_50_O_2_	0.08
45	2,5-Dihydroxybenzaldehyde	Phenols	5.09	C_7_H_6_O_3_	0.07

## Data Availability

The complete lists of DEGs in two bacterial strains are available in NCBI SRA database (https://submit.ncbi.nlm.nih.gov/subs/bioproject/ accessed on 3 December 2021) under the accession number PRJNA830289.

## Supplementary Materials

The following supporting information can be downloaded at: https://www.mdpi.com/article/10.3390/molecules27134308/s1, Table S1: The major altered metabolic pathways in *E. sakazakii* CMCC45401 mediated by the GMPE; Table S2: The major altered metabolic pathways in *S. aureus* ATCC8095 induced by the GMPE; Table S3: Bacterial strains and media used in this study.

## Sample Availability

Samples of the methanol-phase extract from *A. officinarum* Hanceare available from the authors by request.
