# Supplementary material for: Antibacterial Activity and Components of the Methanol-Phase Extract from Rhizomes of Pharmacophagous Plant Alpinia officinarum Hance
Source: Molecules. 2022 Jul 5;27(13):4308. doi: 10.3390/molecules27134308 (PMC9268307; doi:10.3390/molecules27134308)
Supplement: Supplementary file 1 [file molecules-27-04308-s001.zip › molecules-1735867-supplementary.pdf]

Supplementary materials

Table S1. The major altered metabolic pathways in *S. aureus* ATCC8095 induced by the GMPE.

Metabolic Pathway	Gene ID	Fold Change	Gene Description
Ribosome	*EQG65_11565*	2.250	30S ribosomal protein S11
	EQG65_11570	2.398	30S ribosomal protein S13
	EQG65_11575	2.448	50S ribosomal protein L36
	EQG65_11595	2.562	50S ribosomal protein L15
	EQG65_11555	2.681	50S ribosomal protein L17
	EQG65_08510	3.008	50S ribosomal protein L27
	EQG65_14300	3.228	50S ribosomal protein L34
	EQG65_11650	3.257	50S ribosomal protein L29
	EQG65_02705	3.380	30S ribosomal protein S7
	EQG65_11600	3.398	50S ribosomal protein L30
	EQG65_11610	3.444	50S ribosomal protein L18
	EQG65_02380	3.473	50S ribosomal protein L25
	EQG65_08040	3.532	50S ribosomal protein L33
	EQG65_02695	3.550	50S ribosomal protein L7-like protein
	EQG65_11625	3.568	Type Z 30S ribosomal protein S14
	EQG65_02700	3.579	30S ribosomal protein S12
	EQG65_11605	3.595	30S ribosomal protein S5
	EQG65_06500	3.686	30S ribosomal protein S15
	EQG65_11630	3.720	50S ribosomal protein L5
	EQG65_11615	3.733	50S ribosomal protein L6
	EQG65_11525	3.784	30S ribosomal protein S9
	EQG65_11635	3.805	50S ribosomal protein L24
	EQG65_11530	3.826	50S ribosomal protein L13
	EQG65_01735	3.843	30S ribosomal protein S18
	EQG65_11640	3.939	50S ribosomal protein L14
	EQG65_11620	4.009	30S ribosomal protein S8
	EQG65_06310	4.044	30S ribosomal protein S16
	EQG65_10975	4.178	Type B 50S ribosomal protein L31
	EQG65_01725	4.205	30S ribosomal protein S6
	EQG65_11655	4.302	50S ribosomal protein L16
	EQG65_11670	4.304	30S ribosomal protein S19
	EQG65_11645	4.347	30S ribosomal protein S17
	EQG65_11665	4.656	50S ribosomal protein L22
	EQG65_11660	4.665	30S ribosomal protein S3
	EQG65_02655	4.767	50S ribosomal protein L11
	EQG65_11680	4.848	50S ribosomal protein L23
	EQG65_11675	4.930	50S ribosomal protein L2
	EQG65_06405	5.149	30S ribosomal protein S2
	EQG65_08520	5.170	50S ribosomal protein L21
	EQG65_08215	5.198	30S ribosomal protein S20
	EQG65_06325	5.206	50S ribosomal protein L19
	EQG65_11690	5.248	50S ribosomal protein L3
	EQG65_11685	5.264	50S ribosomal protein L4
	EQG65_02660	5.414	50S ribosomal protein L1

	EQG65_11695	5.539	30S ribosomal protein S10
	EQG65_02675	6.426	50S ribosomal protein L7/L12
	EQG65_08665	7.772	50S ribosomal protein L35
	EQG65_08890	7.936	30S ribosomal protein S4
	EQG65_02670	7.942	50S ribosomal protein L10
	EQG65_08660	8.055	50S ribosomal protein L20
Purine metabolism	*EQG65_13860*	0.024	Carbamate kinase
	EQG65_05945	0.059	Carbamate kinase
	EQG65_13755	0.093	Anaerobic ribonucleoside-triphosphate reductase
	EQG65_03735	0.359	Class 1b ribonucleoside-diphosphate reductase subunit α
	EQG65_03740	0.472	Class 1b ribonucleoside-diphosphate reductase subunit β
	EQG65_01860	2.344	IMP dehydrogenase
	EQG65_11585	2.413	Adenylate kinase
	EQG65_11905	2.429	Urease subunit alpha
	EQG65_01865	2.543	Glutamine-hydrolyzing GMP synthase
	EQG65_02425	3.095	Hypoxanthine phosphoribosyltransferase
	EQG65_11895	3.113	Urease subunit gamma
	EQG65_10200	3.237	Adenylosuccinate lyase
	EQG65_08765	3.252	Pyruvate kinase
	EQG65_08460	3.312	Adenine phosphoribosyltransferase
	EQG65_11900	3.957	Urease subunit beta
	EQG65_06890	4.976	GMP reductase
	EQG65_01850	5.085	Xanthine phosphoribosyltransferase
	EQG65_05375	6.772	5-(carboxyamino)imidazole ribonucleotide mutase
	EQG65_05390	7.780	Phosphoribosylformylglycinamidine synthase subunit PurS
	EQG65_05380	9.637	5-(carboxyamino)imidazole ribonucleotide synthase
	EQG65_05385	10.026	Phosphoribosylaminoimidazolesuccinocarboxamide synthase
	EQG65_05425	10.852	Phosphoribosylamine--glycine ligase
	EQG65_05400	13.131	Phosphoribosylformylglycinamidine synthase subunit PurL
	EQG65_05420	13.262	Bifunctional phosphoribosylaminoimidazolecarboxamide formyltransferase/IMP cyclohydrolase
	EQG65_05415	14.297	Phosphoribosylglycinamide formyltransferase
	EQG65_05410	15.616	Phosphoribosylformylglycinamidine cyclo-ligase
	EQG65_00095	16.494	Adenylosuccinate synthase
Alanine, aspartate and glutamate metabolism	*EQG65_13415*	0.070	L-glutamate gamma-semialdehyde dehydrogenase
	EQG65_04810	0.150	NAD-specific glutamate dehydrogenase
	EQG65_11150	0.172	Glutamine--fructose-6-phosphate transaminase (isomerizing)
	EQG65_08830	0.220	Alanine dehydrogenase
	EQG65_07440	0.463	Alanine dehydrogenase
	EQG65_06680	2.208	Type I glutamate--ammonia ligase
	EQG65_02190	5.136	Glutamate synthase subunit β
	EQG65_02185	5.608	Glutamate synthase large subunit
	EQG65_07660	5.839	Asparaginase
	EQG65_05395	13.761	Phosphoribosylformylglycinamidine synthase subunit PurQ
	EQG65_05405	14.775	Amidophosphoribosyltransferase
Pyrimidine metabolism	*EQG65_06130*	15.489	Carbamoyl-phosphate synthetase large subunit
	EQG65_06125	19.069	Carbamoyl-phosphate synthetase small subunit
	EQG65_01440	0.028	Pseudouridine-5'-phosphate glycosidase

	EQG65_11055	0.137	Pyrimidine-nucleoside phosphorylase
	EQG65_06420	2.255	UMP kinase
	EQG65_07655	2.508	(d)CMP kinase
	EQG65_02760	2.707	Deoxynucleoside kinase
	EQG65_08335	3.362	Uridine kinase
	EQG65_10965	3.370	Thymidine kinase
	EQG65_13595	4.843	Quinone-dependent dihydroorotate dehydrogenase
	EQG65_11010	5.962	CTP synthase
	EQG65_06105	8.209	Bifunctional pyr operon transcriptional regulator/uracil phosphoribosyltransferase PyrR
	EQG65_06140	12.374	Orotate phosphoribosyltransferase
	EQG65_06135	12.955	Orotidine-5'-phosphate decarboxylase
	EQG65_06120	22.780	Dihydroorotase
	EQG65_06115	30.276	Aspartate carbamoyltransferase
Fatty acid biosynthesis	*EQG65_00950*	0.073	Long-chain fatty acid--CoA ligase
	EQG65_08315	0.457	Acetyl-CoA carboxylase biotin carboxyl carrier protein subunit
	EQG65_07920	2.312	Acetyl-CoA carboxylase biotin carboxyl carrier protein
	EQG65_06270	2.520	3-oxoacyl-[acyl-carrier-protein] reductase
	EQG65_06265	2.705	[acyl-carrier-protein] S-malonyltransferase
	EQG65_07915	2.771	Acetyl-CoA carboxylase biotin carboxylase subunit
	EQG65_06540	2.847	SDR family NAD(P)-dependent oxidoreductase
	EQG65_04940	3.089	Beta-ketoacyl-[acyl-carrier-protein] synthase II
	EQG65_08780	4.037	Acetyl-CoA carboxylase carboxyltransferase subunit beta
	EQG65_08775	4.062	Acetyl-CoA carboxylase carboxyltransferase subunit alpha
	EQG65_04935	5.502	Ketoacyl-ACP synthase III
Protein export	*EQG65_13950*	0.423	Accessory Sec system translocase SecA2
	EQG65_06305	2.006	Signal recognition particle protein
	EQG65_13970	2.085	Accessory Sec system protein translocase subunit SecY2
	EQG65_01625	2.284	Twin-arginine translocase TatA/TatE family subunit
	EQG65_11590	2.326	Preprotein translocase subunit SecY
	EQG65_01620	2.561	Twin-arginine translocase subunit TatC
	EQG65_10815	2.579	Membrane protein insertase YidC
	EQG65_03855	2.590	Preprotein translocase subunit SecA
	EQG65_04850	3.062	Signal peptidase IB
	EQG65_08470	3.388	Protein translocase subunit SecDF
	EQG65_06095	3.405	Lipoprotein signal peptidase
	EQG65_04845	3.472	Signal peptidase I
Carotenoid biosynthesis	*EQG65_13470*	0.103	Glycosyl-4%2C4'-diaponeurosporenoate acyltransferase
	EQG65_13465	0.125	Diapolycopene oxygenase
	EQG65_13460	0.212	4%2C4'-diaponeurosporenoate glycosyltransferase
	EQG65_13450	0.232	Dehydrosqualene desaturase
	EQG65_13455	0.312	Dehydrosqualene synthase
Arginine biosynthesis	*EQG65_13875*	0.014	Arginine deiminase
	EQG65_13870	0.015	Ornithine carbamoyl transferase
	EQG65_05940	0.042	Ornithine carbamoyl transferase
	EQG65_11195	0.094	Arginase
Aminoacyl-tRNA	*EQG65_08945*	2.034	Tyrosine--tRNA ligase

biosynthesis	*EQG65_02465*	2.052	Lysine--tRNA ligase
	EQG65_08435	2.232	Aspartate--tRNA ligase
	EQG65_09115	2.439	Leucine--tRNA ligase
	EQG65_06195	2.777	Methionyl-tRNA formyltransferase
	EQG65_08575	2.796	Valine--tRNA ligase
	EQG65_08440	2.823	Histidine--tRNA ligase
	EQG65_08110	2.953	Glycine--tRNA ligase
	EQG65_06445	3.241	Proline--tRNA ligase
	EQG65_05770	3.281	Phenylalanine--tRNA ligase subunit beta
	EQG65_10165	4.021	Asp-tRNA (Asn)/Glu-tRNA (Gln) amidotransferase subunit GatC
	EQG65_05765	4.057	Phenylalanine--tRNA ligase subunit alpha
	EQG65_10155	4.146	Asp-tRNA (Asn)/Glu-tRNA (Gln) amidotransferase subunit GatB
	EQG65_10160	4.280	Asp-tRNA (Asn)/Glu-tRNA (Gln) amidotransferase subunit GatA
	EQG65_06075	4.401	Isoleucine--tRNA ligase
Carbon fixation in photosynthetic organisms	*EQG65_08705*	0.013	Type I glyceraldehyde-3-phosphate dehydrogenase
	EQG65_09275	0.032	Phosphoenolpyruvate carboxykinase (ATP)
	EQG65_13195	0.134	Fructose-1%2C6-bisphosphatase
	EQG65_06920	2.036	Transketolase
	EQG65_03980	3.277	Triose-phosphate isomerase
	EQG65_03975	3.496	Phosphoglycerate kinase
	EQG65_03970	3.781	Type I glyceraldehyde-3-phosphate dehydrogenase
	EQG65_11000	3.929	Fructose-bisphosphate aldolase
PTS	*EQG65_01540*	0.021	PTS sugar transporter subunit IIA
	EQG65_01530	0.056	PTS ascorbate transporter subunit IIC
	EQG65_01025	0.084	PTS sugar transporter subunit IIA
	EQG65_01535	0.106	PTS sugar transporter subunit IIB
	EQG65_01035	0.143	PTS galactitol transporter subunit IIC
	EQG65_01030	0.195	PTS galactitol transporter subunit IIB
	EQG65_13910	0.430	PTS mannose transporter subunit IIABC
	EQG65_03560	24.254	PTS fructose transporter subunit IIC
	EQG65_03555	28.586	1-phosphofructokinase

Table S2. The major altered metabolic pathways in *E. sakazakii* CMCC45401 mediated by the GMPE.

Metabolic Pathway	Gene ID	Fold Change	Gene Description
Tryptophan metabolism	*AFK63_10145*	2.043	Hydroperoxidase
	AFK63_04175	2.046	Indolepyruvate decarboxylase
	AFK63_02085	3.317	Peroxidase
ABC transporters	*AFK63_15425*	0.163	Capsule biosynthesis protein
	AFK63_07285	0.204	Nitrate/bicarbonate ABC transporter substrate-binding protein
	AFK63_12140	0.224	Glutamine ABC transporter substrate-bindnig protein
	AFK63_12150	0.237	Glutamine ABC transporter ATP-binding protein
	AFK63_12145	0.255	Glutamine ABC transporter permease
	AFK63_04315	0.295	Heme ABC transporter permease
	AFK63_04505	0.315	Amino acid ABC transporter permease
	AFK63_12390	0.349	Molybdenum ABC transporter ATP-binding protein
	AFK63_04500	0.360	Amino acid ABC transporter permease
	AFK63_04490	0.381	Histidine ABC transporter substrate-binding protein HisJ
	AFK63_16815	0.400	Amino acid ABC transporter permease
	AFK63_16810	0.408	Amino acid ABC transporter substrate-binding protein
	AFK63_15090	0.415	Thiamine ABC transporter substrate-binding protein
	AFK63_16820	0.433	Amino acid ABC transporter permease
	AFK63_04495	0.435	Histidine ABC transporter substrate-binding protein HisJ
	AFK63_12970	1.779	ABC transporter
	AFK63_19755	1.789	Leucine ABC transporter substrate-binding protein
	AFK63_00740	1.799	Maltose transporter
	AFK63_11885	1.821	Putrescine transporter ATP-binding subunit
	AFK63_01455	1.824	Sugar ABC transporter ATP-binding protein
	AFK63_08460	1.829	Choline ABC transporter permease
	AFK63_18595	1.845	Sugar ABC transporter ATP-binding protein
	AFK63_18600	1.868	Ribose ABC transporter permease
	AFK63_18485	2.015	Phosphate ABC transporter substrate-binding protein
	AFK63_01450	2.177	Sugar ABC transporter substrate-binding protein
	AFK63_12985	2.187	ABC transporter substrate-binding protein
	AFK63_00745	2.458	Sugar ABC transporter substrate-binding protein
	AFK63_19775	2.551	Leucine/isoleucine/valine transporter permease subunit
	AFK63_19780	2.573	Leucine/isoleucine/valine transporter ATP-binding subunit
	AFK63_19785	2.765	Leucine/isoleucine/valine transporter ATP-binding subunit
	AFK63_19770	3.149	Branched-chain amino acid transporter permease subunit LivH
	AFK63_19280	3.162	Xylose transporter
	AFK63_06125	3.497	Sugar ABC transporter substrate-binding protein
	AFK63_00750	3.726	Maltose/maltodextrin transporter ATP-binding protein
	AFK63_06130	3.765	L-arabinose transporter ATP-binding protein
	AFK63_19285	3.994	D-xylose transporter subunit XylF
Fructose and mannose metabolism	*AFK63_06860*	1.769	PTS mannose transporter subunit IIAB
	AFK63_05265	1.816	Fructose-bisphosphate aldolase
	AFK63_08160	2.291	Mannose-6-phosphate isomerase
	AFK63_05020	2.405	1-phosphofructokinase
	AFK63_19290	4.281	Xylose isomerase
	AFK63_05425	6.011	Phosphomannomutase
	AFK63_05405	8.932	GDP-fucose synthetase

	AFK63_05400	9.129	GDP-mannose 4%2C6-dehydratase
	AFK63_05420	13.341	Mannose-1-phosphate guanyltransferase
Fatty acid degradation	*AFK63_06955*	1.935	Long-chain fatty acid--CoA ligase
	AFK63_17080	2.113	3-ketoacyl-CoA thiolase
	AFK63_14360	2.388	Acyl-CoA dehydrogenase
	AFK63_17075	2.668	Multifunctional fatty acid oxidation complex subunit alpha
	AFK63_08175	5.512	Alcohol dehydrogenase
Butanoate metabolism	*AFK63_08680*	0.305	Succinate dehydrogenase
	AFK63_12550	1.959	Succinate dehydrogenase
	AFK63_12555	2.020	Succinate dehydrogenase
	AFK63_08185	2.029	Pyruvate-flavodoxin oxidoreductase
	AFK63_08940	2.209	3-oxoacyl-ACP reductase
	AFK63_07645	3.368	Formate acetyltransferase
Glycosphingolipid biosynthesis	*AFK63_17760*	2.158	α-galactosidase
	AFK63_12700	2.290	β-N-acetylhexosaminidase
Starch and sucrose metabolism	*AFK63_06140*	1.901	Trehalose phosphatase
	AFK63_19875	1.921	Glycogen branching protein
	AFK63_13750	2.059	Maltodextrin glucosidase
	AFK63_19935	2.077	Maltose phosphorylase
	AFK63_06145	2.258	Trehalose-6-phosphate synthase
	AFK63_08030	2.335	β-phosphoglucomutase
	AFK63_01700	2.383	PTS cellobiose transporter subunit IIC
	AFK63_09960	2.699	β-D-glucoside glucohydrolase
	AFK63_19260	3.972	α-amylase
	AFK63_01705	4.086	PTS lactose transporter subunit IIB
	AFK63_15600	4.313	PTS cellobiose transporter subunit IIC
	AFK63_02320	7.040	6-phospho-beta-glucosidase
Lysine degradation	*AFK63_16040*	1.807	Putrescine--2-oxoglutarate aminotransferase
	AFK63_12535	1.900	Dihydrolipoamide succinyltransferase
	AFK63_16660	2.441	Succinate-semialdehyde dehydrogenase
Other glycan degradation	*AFK63_14170*	2.011	β-D-galactosidase
Benzoate degradation	*AFK63_08975*	2.202	Tautomerase PptA

Table S3. Bacterial strains and media used in this study.

Bacterial strain	Culture medium	Source
Aeromonas hydrophila	TSB	LS-SHOU, China
Aeromonas hydrophila ATCC35654	TSB	ATCC, United States
Enterobacter cloacae ATCC13047	TSB	Biobw, China
Enterobacter cloacae	LB	LS-SHOU, China
Enterobacter sakazakii CMCC45401	TSB	Biobw, China
Escherichia coli ATCC8739	TSB	Biobw, China
Escherichia coli K12	TSB	IIM, China
Escherichia coli ATCC25922	LB	ATCC, United States
Listeria monocytogenes ATCC19115	BHI	Biobw, China
Pseudomonas aeruginosa ATCC9027	TSB	Biobw, China
Pseudomonas aeruginosa ATCC27853	TSB	Biobw, China
Salmonella enterica subsp. *enterica* (*ex* Kauffmann and Edwards) Le Minor and Popoff serovar Choleraesuis ATCC13312	TSB	ATCC, United States
Salmonella enterica subsp. *enterica* (*ex* Kauffmann and Edwards) Le Minor and Popoff serovar Vellore ATCC15611	TSB	ATCC, United States
Salmonella paratyphi-A CMCC50093	TSB	GCCC, China
Shigella dysenteriae CMCC51252	TSB	GCCC, China
Shigella flexneri CMCC51572	TSB	GCCC, China
Shigella flexneri ATCC12022	TSB	ATCC, United States
Shigella flexneri CMCC51574	TSB	GCCC, China
Shigella sonnei ATCC25931	TSB	ATCC, United States
Shigella sonnet CMCC51592	TSB	GCCC, China
Staphylococcus aureus GIM1.160	TSB	GCCC, China
Staphylococcus aureus ATCC25923	TSB	ATCC, United States
Staphylococcus aureus ATCC8095	TSB	ATCC, United States
Staphylococcus aureus ATCC29213	TSB	ATCC, United States
Staphylococcus aureus ATCC6538	TSB	ATCC, United States
Staphylococcus aureus GIM1.441	TSB	LS-SHOU, China
Vibrio alginolyticus	TSB	LS-SHOU, China
Vibrio alginolyticus ATCC17749	TSB	ATCC, United States
Vibrio alginolyticus ATCC33787	TSB	ATCC, United States
Vibrio fluvialis ATCC33809	Marine 2216	ATCC, United States
Vibrio harvey ATCC BAA-1117	Marine 2216	ATCC, United States
Vibrio harveyi ATCC33842	Marine 2216	ATCC, United States
Vibrio metschnikovii ATCC700040	Marine 2216	ATCC, United States
Vibrio mimicus bio-56759	TSB	Biobw, China
Vibrio parahaemolyticus ATCC17802	TSB	ATCC, United States
Vibrio parahaemolyticus B3-13	TSB	LS-SHOU, China
Vibrio parahaemolyticus B4-10	TSB	LS-SHOU, China
Vibrio parahaemolyticus B5-29	TSB	LS-SHOU, China
Vibrio parahaemolyticus B9-35	TSB	LS-SHOU, China
Vibrio vulnificus ATCC27562	TSB	Biobw, China
Vibrio vulnificus	TSB	LS-SHOU, China

ATCC: American Type Culture Collection, United States; GCCC, Guangdong Culture Collection Center, Guangzhou, China; IIM, Institute of Industrial Microbiology, Shanghai, China; LS-SHOU, Laboratory stock, Shanghai Ocean University, Shanghai, China.
